# The effect of post mastectomy radiation therapy on breast reconstruction with and without acellular dermal matrix: a systematic review and meta-analysis protocol

**DOI:** 10.1186/s13643-019-0958-z

**Published:** 2019-02-21

**Authors:** Amy M. Chung, Michael J. Stein, Ammara Ghumman, Jing Zhang

**Affiliations:** 1 Faculty of Medicine, University of Ottawa, Ottawa, Canada; 2 Division of Plastic and Reconstructive Surgery, Univeristy of Ottawa, Ottawa, Ontario Canada; 3 Department of Surgery, The Ottawa Hospital - Civic Campus, 1053 Carling Avenue Box 213, Ottawa, ON K1Y 4E9 Canada

## Abstract

**Background:**

The widespread implementation of acellular dermal matrix (ADM) has broadened the reconstructive repertoire for alloplastic breast reconstruction. ADM’s role in the context of postoperative radiation therapy remains unclear. The present review will evaluate whether ADM reduces complication rates in patients undergoing post-mastectomy radiation therapy (PMRT).

**Methods:**

A healthcare librarian assisted in performing a search strategy of electronic databases MEDLINE (via Ovid), EMBASE, and CENTRAL. A combination of the keywords and Medical Subject Headings (MESH) to describe the various commercially available ADMs and terms for radiation therapy will be used. The search strategy will identify patients undergoing postoperative radiation following implant-based breast reconstruction and compare outcomes between those with and without ADM. Extracted data will include patient demographics, intraoperative data, and postoperative complications. Data on patient satisfaction and resource utilization will also be extracted if available. The references of selected works will be reviewed for additional studies meeting study criteria. Only peer-reviewed papers written in English will be included. The study data will be assessed for risk of bias and heterogeneity. Providing that sufficient studies can be identified, a meta-analysis will be performed. This review has been registered with PROSPERO (CRD42017056495).

**Conclusions:**

To date, the short- and long-term performance of ADM in the context of postoperative radiation remains unclear. The objective of the present review will be to critically evaluate the literature with the intention of improving postoperative outcomes in the context of mastectomy and radiation.

**Electronic supplementary material:**

The online version of this article (10.1186/s13643-019-0958-z) contains supplementary material, which is available to authorized users.

## Background

Acellular dermal matrix (ADM) is a biointegrative scaffold derived from cadaveric dermis devoid of cellular components. The use of ADM has been transformative in the context of breast reconstruction by providing support and vascularized coverage to the reconstructed breast. They have been shown to lower the risk of capsular contracture, decrease implant migration, increase fill volumes, and improve aesthetic outcomes [1–3], albeit with an increased risk for seroma formation and infection [4, 5].

PMRT applied to implant-based breast reconstruction has been associated with a high risk of reconstructive failure and capsular contracture [6]. Complications have been reported to be up to four times higher when ADM is used in the context of radiation [7]. Histologically, radiation therapy results in the inhibition of matrix metalloproteinase-1 and an increase in the pro-inflammatory cytokines TGF-β, TNF-α, and IFN-γ leading to dysregulation of neovascularization and disordered tissue remodeling and integration [3]. In a 2015 histologic study, Mckatyn et al. [8] demonstrated less integration of ADM in patients exposed to PMRT. Biopsies of these patients revealed less vascular penetrance, cellularization, and constructive remodeling of the ADM. Cavallo et al. further demonstrated similar results with preoperative radiation, which resulted in poor cellular and vascular infiltration of the ADM [9].

Others report the contrary. Sbitany et al. [10] demonstrated that ADM was associated with a lower risk of infection and tissue expander/implant exposure than those not using it. Similarly, Peled et al. [11] demonstrated that the increased coverage ADM provides lead to a lower rate of expander-implant failure after PMRT compared to partial coverage. Another study evaluating pre-reconstruction radiation found no difference in complication rates with ADM use [12–14].

To date, it is clear that no consensus exists with respect to the use of ADM in the context of one or two stage alloplastic breast reconstruction. The objective of the present study is to compare postoperative outcomes between patients with and without ADM in the context of preoperative and postoperative radiation.

## Methods

### Search strategy

A detailed literature search will be conducted of the electronic databases MEDLINE (via Ovid), EMBASE, and CENTRAL from inception to present. A combination of the keywords and Medical Subject Headings will be used including “Acellular Dermis,” “AlloDerm,” “Regenerative Tissue Matrix,” “DermACELL,” “Flex HD,” “DermaMatrix,” “AlloMax,” “SurgiMend,” “Radiation Oncology,” “Radiotherapy,” “Irradiation,” “Breast,” and “Mammoplasty.” The search strategy will be performed by two independent reviewers and compared for consistency (AC and AG). The references of selected works will be assessed by each reviewer to identify additional articles that meet inclusion criteria.

### Study criteria

The types of study to be included are randomized control trials, quasi-randomized studies, cohort studies, case-control, and case series. The gray literature will be searched using the FDA database, ClinicalTrials.gov, and ProQuest Dissertations. Peer-reviewed papers written in English will be included and other languages if translation was provided. Publications involving animal subjects and case-series of less than 10 patients will be excluded. Inclusion criteria consist of female patients 18 years of age or older who underwent alloplastic breast reconstruction and radiation therapy. The primary exposure is ADM use, and studies should compare reconstructions with and without ADM. Specifically, we will not include studies in which all patients were reconstructed with ADM and in which the groups were compared based on radiation status. Table 1 outlines the structure of the study inclusion criteria. The primary outcome of interest is reconstruction failure defined as implant loss. Data on postoperative complications such as infection, seroma, hematoma, dehiscence, capsular contracture, and skin necrosis as defined in Table 2 will be collected and analyzed separately. Lastly, patient-reported outcomes will be included when available using the BREAST-Q questionnaire.

**Table 1:** PICO format of studies for inclusion in the structured literature review

Population	Women > 18 years of age undergoing implant-based breast reconstruction AND radiation therapy
Intervention	ADM use in reconstructive surgeries
Comparison	Reconstructions performed without ADM
Outcome	Reconstruction failure

**Table 2:** Definition of postoperative complications

Complication	Definition
Superficial surgical site infection (SSI)	Infection at the surgical site requiring oral antibiotics
Deep superficial site infection (SSI)	Infection at the surgical site requiring intravenous antibiotics
Seroma	A collection of clear fluid in the breast
Hematoma	A collection of blood in the breast
Dehiscence	Opening of the wound along the surgical incision
Capsular contracture	The formation of disruptive scar formation surrounding the implant capsule of Baker Grade III or IV
Skin necrosis	Cell death of the mastectomy flap or skin surrounding the surgical site

### Screening

Search results will be entered into the latest version of EndNote (Clarivate Analytics), and duplicates will be removed. The search results will be screened by two independent reviewers (AC and AG) in a two-stage process, first, based the title and abstract, and second as a review of the full article. Disagreements will be resolved by consulting a third, more senior reviewer. The justification for exclusion from the review will be documented.

### Data extraction

This literature review will be conducted in accordance with the PRISMA-P statement to ensure comprehensiveness and transparency [15]. Data will be extracted using predesigned forms. In the event of missing data, the corresponding author of the study will be contacted (Additional files 1 and 2).

### Data analysis

The program Review Manager (RevMan) 5.1 will be used for data analysis and to tabulate the findings. The unit of analysis will be by patient as opposed to by breast. For studies reporting outcomes by breast, the data will be converted to reflect the complications by patient where possible. Breast reconstruction reflects a heterogenous population based on whether it was immediate or delayed and one or two stages. The analysis will seek to group studies with the same reconstructive timing and stages together. The primary outcome will be reconstruction failure. Data will be analyzed and grouped based on study design. The study outcomes will be reported as proportions and analyzed using odds ratios (OR). We will evaluate reconstruction failure and specific complications where applicable. Data will be summarized using Forest plots. The *I*^2^ test will be used to evaluate statistical heterogeneity [16]. If there is low-moderate heterogeneity detected (*I*^2^ < 50%), we will perform a fixed-effects meta-analysis [17]. The Mantel-Haenszel method will be used to compute a weighted odds ratio. If there is substantial heterogeneity (*I*^2^ > 50%) or clinical heterogeneity, we will attempt to determine explanations for this and will apply a random-effects model for analysis [18] A sensitivity analysis will be performed by removing trials that are outliers, in order to determine the degree to which the overall outcomes were influenced by contributions to the heterogeneity. Given the multitude of reconstructive options available to breast cancer patients, we anticipate considerable heterogeneity among the studies.

Publication bias will be assessed graphically using a funnel plot where the treatment effect is plotted against a measure of study size [19]. Individual, randomized studies will be assessed by the reviewers using the Cochrane Risk of Bias tool [24] [20]. Non-randomized studies will be assessed using the Risk Of Bias In Non-randomized Studies-of Interventions (ROBINS-I) Tool [21]. Each outcome will be assigned a GRADE score. The ability to perform a meta-analysis will depend on the number of articles identified from the search.

#### Limitations

The present review is limited by the heterogeneity in the literature given the variation in radiation timing and multitude of reconstructive options available to women. Furthermore, as our search was limited to radiated patients it is possible to miss data if the data on ADM and radiation is part of a subanalysis of a larger study.

## Discussion

Since its introduction in 1994, it has become clear that ADM has made a significant positive impact on patients undergoing alloplastic breast reconstruction. Despite this, the full scope of its performance, particularly in the context of radiation, remains unclear. The present review hopes to determine the effect of ADM on outcomes in patients who underwent immediate breast reconstruction and radiation therapy.

## Conclusions

The present review will help inform guidelines regarding the use of ADM in patients receiving preoperative and postoperative radiation.

## Additional files

Additional file 1: Search Strategy for electronic database search of MEDLINE (via Ovid), EMBASE and CENTRAL. (DOCX 15 kb)

Additional file 2: PRISMA-P Checklist for study. (DOCX 9 kb)

## Abbreviations

ADM: Acellular dermal matrix
PMRT: Post mastectomy radiation therapy
PRISMA-P: Preferred Reporting Items for Systematic Reviews and Meta-Analysis Protocol
