# Supplementary material for: The effect of post mastectomy radiation therapy on breast reconstruction with and without acellular dermal matrix: a systematic review and meta-analysis protocol
Source: Syst Rev. 2019 Feb 21;8:58. doi: 10.1186/s13643-019-0958-z (PMC6383242; doi:10.1186/s13643-019-0958-z)
Supplement: Supplementary file 1 — Search Strategy for electronic database search of MEDLINE (via Ovid), EMBASE and CENTRAL. (DOCX 15 kb) [file 13643_2019_958_MOESM1_ESM.docx]

**Additional File 1: Search Strategy**

Database: Epub Ahead of Print, In-Process & Other Non-Indexed Citations, Ovid MEDLINE(R) Daily and Ovid MEDLINE(R) (1946 to Present)

Search Strategy:

1 Acellular Dermis/ (484)

2 Acellular Derm*.ti,ab. (1398)

3 Alloderm*.ti,ab. (506)

4 Regenerative Tissue Matri*.ti,ab. (33)

5 DermACEL*.ti,ab. (9)

6 Flex HD*.ti,ab. (4)

7 DermaMatrix*.ti,ab. (20)

8 AlloMax*.ti,ab. (16)

9 SurgiMend*.ti,ab. (10)

10 or/1-9 (1864)

11 Radiation Oncology/ (3229)

12 Radiotherapy/ or Radiotherapy, Conformal/ or Radiotherapy, Computer-Assisted/ or Radiotherapy, Image-Guided/ or Radiotherapy Planning, Computer-Assisted/ or Radiotherapy, Adjuvant/ (77230)

13 Radiat*.ti,ab. (311756)

14 Radiotherap*.ti,ab. (136248)

15 irradiat*.ti,ab. (209939)

16 or/11-15 (571473)

17 10 and 16 (152)

18 Mammaplasty/ (9119)

19 Breast/ (34526)

20 Mammaplast*.ti,ab. (2005)

21 breast reconstruction*.ti,ab. (6152)

22 Breast*.ti,ab. (361060)

23 or/18-22 (370752)

**24 17 and 23 (97)**

Database: Embase Classic+Embase (1947 to 2016 August 04)

Search Strategy:

1 acellular dermal matrix/ (1064)

2 (acellular adj2 derm*).tw. (1820)

3 alloderm.tw. (511)

4 1 or 2 or 3 (2379)

5 exp breast reconstruction/ (17082)

6 exp breast implant/ (1462)

7 (breast and (reconstruct* or implant*)).tw. (20406)

8 mammaplast*.tw. (2336)

9 5 or 6 or 7 or 8 (28354)

10 4 and 9 (474)

11 exp radiotherapy/ (515775)

12 (radiotherapy or radiation therapy or irradiat*).tw. (517808)

13 radiation injury/ (51980)

14 radiation dermatitis/ (3282)

15 (radiat* or radiodermatitis).tw. (428248)

16 or/11-15 (933278)

**17 10 and 16 (134)**
