# Supplementary material for: The effect of post mastectomy radiation therapy on breast reconstruction with and without acellular dermal matrix: a systematic review and meta-analysis protocol
Source: Syst Rev. 2019 Feb 21;8:58. doi: 10.1186/s13643-019-0958-z (PMC6383242; doi:10.1186/s13643-019-0958-z)
Supplement: Supplementary file 2 — PRISMA-P Checklist for study. (DOCX 9 kb) [file 13643_2019_958_MOESM2_ESM.docx]

**PRISMA-P (Preferred Reporting Items for Systematic review and Meta-Analysis Protocols) 2015 checklist**

| **Section and topic** | **Item No** | **Checklist item** |  |
| --- | --- | --- | --- |
| **ADMINISTRATIVE INFORMATION** | | |  |
| Title: |  |  |  |
| Identification | 1a | Identify the report as a protocol of a systematic review | The title is identified as a protocol of a systematic review. |
| Update | 1b | If the protocol is for an update of a previous systematic review, identify as such | This work is not an update to a previous review. |
| Registration | 2 | If registered, provide the name of the registry (such as PROSPERO) and registration number | This review has been registered with PROSPERO (CRD42017056495). |
| Authors: |  |  |  |
| Contact | 3a | Provide name, institutional affiliation, e-mail address of all protocol authors; provide physical mailing address of corresponding author | Detailed in manuscript- ‘Author Details” |
| Contributions | 3b | Describe contributions of protocol authors and identify the guarantor of the review | Detailed in manuscript- ‘Author Details” |
| Amendments | 4 | If the protocol represents an amendment of a previously completed or published protocol, identify as such and list changes; otherwise, state plan for documenting important protocol amendments | This is an original protocol. Significant protocol amendments will be documented under the PROSPERO registry. |
| Support: |  |  |  |
| Sources | 5a | Indicate sources of financial or other support for the review | There is no funding associated with this study. |
| Sponsor | 5b | Provide name for the review funder and/or sponsor | Not applicable |
| Role of sponsor or  funder | 5c | Describe roles of funder(s), sponsor(s), and/or institution(s), if any, in developing the protocol | Not applicable |
| **INTRODUCTION** | | |  |
| Rationale | 6 | Describe the rationale for the review in the context of what is already known | Detailed in manuscript- ‘Background’ |
| Objectives | 7 | Provide an explicit statement of the question(s) the review will address with reference to participants, interventions, comparators, and outcomes (PICO) | Detailed in manuscript- ‘Background’ |
| **METHODS** | | |  |
| Eligibility criteria | 8 | Specify the study characteristics (such as PICO, study design, setting, time frame) and report characteristics (such as years considered, language, publication status) to be used as criteria for eligibility for the review | Detailed in manuscript- ‘Methods – Section Criteria” |
| Information sources | 9 | Describe all intended information sources (such as electronic databases, contact with study authors, trial registers or other grey literature sources) with planned dates of coverage | Detailed in manuscript- ‘Methods – Study Criteria” |
| Search strategy | 10 | Present draft of search strategy to be used for at least one electronic database, including planned limits, such that it could be repeated | Detailed in Additional File 1 |
| Study records: |  |  |  |
| Data management | 11a | Describe the mechanism(s) that will be used to manage records and data throughout the review | Detailed in manuscript- “Methods – Screening” |
| Selection process | 11b | State the process that will be used for selecting studies (such as two independent reviewers) through each phase of the review (that is, screening, eligibility and inclusion in meta-analysis) | Detailed in manuscript- “Methods – Screening” |
| Data collection process | 11c | Describe planned method of extracting data from reports (such as piloting forms, done independently, in duplicate), any processes for obtaining and confirming data from investigators | Detailed in manuscript- “Methods – Data Extraction” |
| Data items | 12 | List and define all variables for which data will be sought (such as PICO items, funding sources), any pre-planned data assumptions and simplifications | Detailed in manuscript- “Methods – Data Extraction” |
| Outcomes and prioritization | 13 | List and define all outcomes for which data will be sought, including prioritization of main and additional outcomes, with rationale | Detailed in manuscript- ‘Methods – Data Extraction” section |
| Risk of bias in individual studies | 14 | Describe anticipated methods for assessing risk of bias of individual studies, including whether this will be done at the outcome or study level, or both; state how this information will be used in data synthesis | Detailed in manuscript- ‘Methods – Data Analysis” section |
| Data synthesis | 15a | Describe criteria under which study data will be quantitatively synthesised | Detailed in manuscript- ‘Methods – Data Analysis” section |
|  | 15b | If data are appropriate for quantitative synthesis, describe planned summary measures, methods of handling data and methods of combining data from studies, including any planned exploration of consistency (such as I^2^, Kendall’s τ) | Detailed in manuscript- ‘Methods – Data Analysis” section |
|  | 15c | Describe any proposed additional analyses (such as sensitivity or subgroup analyses, meta-regression) | Detailed in manuscript- ‘Methods – Data Analysis” section |
|  | 15d | If quantitative synthesis is not appropriate, describe the type of summary planned | Detailed in manuscript- ‘Methods – Data Analysis” section |
| Meta-bias(es) | 16 | Specify any planned assessment of meta-bias(es) (such as publication bias across studies, selective reporting within studies) | Detailed in manuscript- ‘Methods – Data Analysis” section |
| Confidence in cumulative evidence | 17 | Describe how the strength of the body of evidence will be assessed (such as GRADE) | Detailed in manuscript- ‘Methods – Data Analysis” section |

*From: Shamseer L, Moher D, Clarke M, Ghersi D, Liberati A, Petticrew M, Shekelle P, Stewart L, PRISMA-P Group. Preferred reporting items for systematic review and meta-analysis protocols (PRISMA-P) 2015: elaboration and explanation. BMJ. 2015 Jan 2;349(jan02 1):g7647.*
